# Prescription for antidepressant in reducing future alcohol-related readmission in patients suffering from depression and alcohol use disorder: a retrospective medical record review

**DOI:** 10.1186/s13011-015-0045-y

**Published:** 2015-12-21

**Authors:** Patrick Chan, Katie Yomen, Jennifer Turcios, Mark Richman

**Affiliations:** University of California Los Angeles Medical Center, 757 Westwood Plaza, Room B531, Los Angeles, CA USA; Kaiser Permanente Baldwin Park, 1011 Baldwin Park Blvd, Baldwin Park, CA USA; Department of Emergency Medicine, North Shore-LIJ Long Island Jewish Medical Center, 270-05 76th Ave, New Hyde Park, NY 11040 USA; Western University of Health Sciences, 309 E. 2nd St, Pomona, CA 91766 USA

**Keywords:** Alcohol-use disorder, Major depressive disorder, Antidepressant, Alcohol-related readmission

## Abstract

**Background:**

Patients suffering from major depressive disorder are more likely to suffer from alcohol use disorder. The data is inconclusive for the effectiveness of antidepressant treatment of patients suffering from both illnesses in regards to improving sobriety and reducing alcohol-related healthcare expenses such as hospitalizations. The objective of this study is to determine if a new prescription of an antidepressant upon inpatient discharge is associated with a reduction in the number of future acute alcohol-related hospital readmissions to the same institution in patients suffering from major depressive disorder and alcohol-use disorder.

**Methods:**

A retrospective, medical record review study was conducted at a publicly-supported hospital in Sylmar, CA. A query was performed for adult patients admitted between 1/1/2005–12/31/2013 who had ICD-9 codes for both alcohol-use disorder and depression. Index admission was the first hospitalization in which the patient was currently consuming alcohol and had depression as identified by physician documentation as a problem. Acute alcohol-related admissions were those for alcohol intoxication or withdrawal (indicating current alcohol use). Patients were excluded if they were receiving an antidepressant on index admission, <18 years old, no patient data available, or not currently consuming alcohol; 139 patients met inclusion criteria. Multivariate logistical regression analysis was performed on the primary predictive variable of discharge prescription of an antidepressant along with other independent variables for alcohol readmissions: homelessness, family history of alcohol use disorder, and smoking.

**Results:**

Discharging patients with a prescription of an antidepressant was not associated with a reduction in acute alcohol-related readmission. There was no difference in acute alcohol-related readmissions between patients discharged with (44.6 %) versus without (47.0 %) a prescription for an antidepressant (*p* = 0.863). The median number of days between index admission and first readmission for those discharged on an antidepressant was 141 days while those who were not was 112 days (*p* = 0.284).

**Conclusion:**

Discharging patients suffering from both alcohol-use disorder and major depressive disorder with a prescription for an antidepressant is not associated with a reduction in future readmissions, nor significantly increase the number of days to readmission. The study does not support the concept of antidepressants in reducing acute alcohol-related readmissions.

## Background

Major depressive disorder (MDD) and alcohol abuse continue to plague patients and society. In the United States, MDD is a mood disorder affecting millions of people with a lifetime prevalence of 16.2, and 7.6 % Americans suffering from moderate to major depressive symptoms within the past two weeks [1, 2]. The National Institute on Alcohol Abuse and Alcoholism estimated that 18 million Americans suffered from alcohol use disorder (AUD) in 2007, with a 12-month prevalence of 14 % and life prevalence of 29 % [2, 3]. Futhermore, MDD and AUD co-occur at high rates [4]. The National Institute on Alcohol Abuse and Alcoholism’s National Epidemiologic Survey on Alcohol and Related Conditions (NESARC) indicated that patients who suffer from MDD are more likely to suffer from AUD [3], and conversely, those who suffer from alcohol use disorder have a 12 month prevalence of MDD of 16.4 % [5]. For patients suffering from either MDD or AUD, they face numerous challenges in improving their health [6]. For patients who suffer from MDD and AUD concomitantly, the situation becomes much more daunting. Alarmingly, it has been estimated that only 6 % of patients suffering from mental disorders and substance abuse receive treatment from proper medical or mental health care providers in the past six months [7]. The abuse of alcohol is costly, estimated around $25 billion due to health care, crime, and lost productivity [8].

Treatment for AUD is generally initiated during alcohol withdrawal or hospitalization due to an alcohol-related complication. Pharmacotherapeutic options for the treatment of AUD include naltrexone, disulfiram, and acamprosate, in addition to psychosocial treatment [9, 10]. Naltrexone and acamprosate act as deterrents to alcohol consumption. Effective MDD treatment involves both psychotherapy and pharmacotherapy. Pharmacologic options for depression treatment are selective serotonin reuptake inhibitors (SSRIs), serotonin norepinephrine reuptake inhibitors (SNRIs), tricyclic antidepressants (TCAs), and monoamine oxidase inhibitors (MAOIs). While the efficacy between the classes of SSRIs and TCAs is similar, SSRIs are frequently prescribed due to a favorable side-effect profile and fewer drug interactions compared to TCAs. One concern with SSRIs is the risk of serotonin syndrome, a life-threatening adverse reaction which may cause hyperthermia, mental confusion, and myoclonus. MAOIs have largely been avoided due to drug-drug and drug-food interactions.

There is a link between MDD and AUD. Depression is a strong predictor for first incidence of AUD [11]. Treatment of patients diagnosed with both MDD and AUD are less successful than patients with MDD or AUD only [12, 13]. Consensus clinical practice guidelines from the American Psychiatric Association are available for the treatment of AUD [14] and MDD [15] individually. The current data is inconclusive for the effectiveness of antidepressant treatment of patients suffering from both illnesses in regards to improving sobriety and reducing alcohol-related healthcare expenses such as hospitalizations. Mixed results from clinical trials have been reported in the treatment of patients suffering from concurrent MDD and AUD. Patients treated with chlordiazepoxide and imipramine [16, 17], nefazodone [18], sertraline [19, 20], citalopram [21], and TCAs [22] were unsuccessful in reducing alcohol consumption. In contrast, other trials using desipramine [23], fluoxetine [24], sertraline [25], and nefazodone [26] demonstrated a decrease in alcohol use amongst patients. Thus, it is unclear whether antidepressant use in patients suffering from alcohol and depression is effective in improving actual sobriety. A meta-analysis of 11 placebo-controlled randomized trials conducted by Iovieno, et. al. on the treatment of co-occurring depression and alcohol dependence showed that imipramine, desipramine, and nefazodone were most effective for reducing depressive symptoms; however, the data on the efficacy of antidepressants on sobriety was inconclusive due to the limited number of studies analyzing this effect [27].

In recent years there has been a shift in treatment paradigm. More recent studies have employed combination therapy with medications from different pharmacological classes. Patients treated with memantine and escitalopram [28] or aripiprazole and escitalopram [29] showed better outcomes than patients treated with only escitalopram. Both serotonin and dopamine neurotransmitter systems have demonstrated dysfunctional neuromechanisms in patients suffering from alcohol dependence [30]. Aripiprazole is a partial antagonist for dopamine receptors in addition to being an agonist at 5-HT_2A_ (5-hydroxytryptamine; serotonin) receptor. This is the rationale for the addition of aripiprazole to the treatment of patients who are suffering from both MDD and AUD. A study by Pettinati et al. demonstrated higher alcohol abstinence rates in patients treated with both sertraline and naltrexone [31]. However, polytherapy may expose patients to a greater number of side effects or drug interactions.

Studies have identified several risk factors for alcohol-related admissions [32]. A prospective cohort trial focused on risk factors for alcohol-related outcomes such as hospitalization and deaths. Males and social factors such as marital status (single or divorced/widowed), low socioeconomic status, and patients who consumed higher amounts of alcohol per week or cigarettes per day, were associated with an increased risk [32, 33]. Homelessness and family history of alcohol-use disorder were identified to also elevate the risk of alcohol consumption, resulting in an increased risk in the number of alcohol-related admissions [34].

Because of the limited pharmacological options available for the treatment of AUD, it has become pivotal to examine if any of the currently available medications may have an effect in reducing alcohol-related readmissions in patients who are suffering from both MDD and AUD.

## Objectives

This retrospective study aims to add to the literature regarding whether a newly initiated prescription for an antidepressant in patients suffering from depression and alcohol-use disorder is related to a reduced rate of subsequent acute alcohol-related hospital readmissions. A decrease in the number of these readmissions would lower the high economic burden caused by AUD. Clinical evidence of effectiveness demonstrated by antidepressants would provide physicians another compelling indication for prescribing antidepressants to patients with both conditions upon discharge.

## Methods

### Setting

A retrospective, medical record review study conducted at Olive View- University of California, Los Angeles Medical Center (OVMC), a 377-licensed bed, publicly-supported, academic teaching hospital located in Sylmar, CA. It is staffed to 200 beds, serves a medically-indigent population, and functions as a teaching hospital without cardiothoracic, neurosurgical, or inpatient orthopedic services. Olive View’s Emergency Department has approximately 55,000 annual visits, and the hospital admits approximately 15,000 patients annually.

### Study design and patients

A query was conducted to identify patients admitted to the hospital between 1/1/2005-12/31/2013 with an ICD-9 code for both alcohol-use disorder and depression prior to, or at the time of, admission. Patients were excluded if they were currently receiving an antidepressant on index admission, <18 years old, patient data was not available, or if the patient stated that they did not consume alcohol within 72 h of admission as noted in the history and physical in medical records. A total of 139 patients were included for electronic medical record review by two independent reviewers. Patient information was collected at both index admission and subsequent hospital readmissions. Index admission was defined as the first hospitalization in which the patient was currently consuming alcohol and had current depressive symptoms. Current consumption of alcohol was defined as having an alcoholic beverage within 72 h of hospital admission. Patients were considered to have current depressive symptoms if there were any physician documentation during index admission of depression as an active problem. Subsequent readmissions were considered if the discharge diagnosis documented acute intoxication of alcohol, alcohol withdrawal, or acute alcohol-related complication (altered mental status related to alcohol consumption, cirrhosis, alcoholic cardiomyopathy, and gastritis) as noted during the medical record review. During the index admission, the following patient data was collected: demographics, social history (substance abuse disorder and AUD), length of stay (LOS), clinical information, co-morbid disease states, and medication history (specifically antidepressant use prior to admission, during hospitalization, and at discharge). Selective serotonin reuptake inhibitors (SSRI’s; fluoxetine, fluvoxamine, sertraline paroxetine, escitalopram, and citalopram), tricyclic antidepressants (TCA’s; amitriptyline, clomipramine, desipramine, doxepin, imipramine, and nortriptyline), atypical antidepressants (bupropion, duloxetine, venlafaxine, mirtazapine, and trazodone), and monoamine oxidase inhibitors (MAOI’s; phenelzine, tranylcypromaine, and selegiline) were considered prescriptions if noted specifically in medical records as indicated for depression. Literature-identified demographic risk factors for alcohol-related hospital admissions (homelessness, smoking, and family history of AUD) were also recorded. At each subsequent hospital readmission, data such as LOS, antidepressant use before, during, and after discharge, and current alcohol consumption were noted. The primary analysis is focused on whether a prescription for antidepressants is associated with a reduced rate of alcohol-related readmissions in patients suffering from depression and AUD. Secondary outcomes included differences in LOS or intervals between readmissions. This study was IRB approved by Western University of Health Sciences and Olive-View Medical Center.

### Statistical analysis

The primary independent variable for the model is a prescription for antidepressant upon discharge. Other independent variables included in the model were the following demographic variables: gender, homelessness, current smoker, and family history of AUD. The Chi-square and Fisher’s Exact Tests were used to analyze baseline characteristics. The Mann–Whitney test was used to analyze non-normally distributed continuous data such as LOS and time between index admission and first readmission. A multivariate logistic regression analysis with a dichotomous outcome of being readmitted was performed by SPSS (Version 22.0) to analyze the predictive effects of each independent variable and reported as odds ratios and 95 % confidence intervals. All independent variables were included in the model simultaneously. Two-tailed p values were considered, a priori, statistically significant at p < 0.05.

## Results

Baseline characteristics of the 139 patients that were included (discharged on an antidepressant, *n* = 56; discharged without an antidepressant, *n* = 83) in the study are shown in Table 1. The average age was 47 years old in both groups (SD = 9 in those discharged with an antidepressant; SD = 8 in those discharged without an antidepressant); approximately 70 % of patients were male. Patients who had a history of antidepressant use, but not at the time of index admission, were more likely to be discharged with a prescription for an antidepressant. No difference in social factors such as smoking, family history of AUD, and current illicit substance abuse was found between both groups at baseline. The most common comorbidity found among those discharged with versus those discharged without an antidepressant was cirrhosis. There was no statistical significance found between both groups in the number of comorbidities (Table 1). Alcohol-related reasons prompting index admission and readmissions are listed in Table 2.

**Table 1:** Patient characteristics

Baseline Characteristics	Discharged with Antidepressant (*n* = 56)	Discharged without Antidepressant (*n* = 83)	P-value (d.f.)	Test statistic
Gender - Male, n (%)*	40 (71 %)	58 (70 %)	0.84 (1)	0.04
Age in years (mean ± SD)***	47 (±9)	47 (±8)	0.77 (137)	1.24
Homeless, n (%)*	8 (14 %)	19 (23 %)	0.21 (1)	2.58
Current smoker, n (%)*	18 (32 %)	26 (31 %)	0.91 (1)	0.01
Family history of alcohol abuse, n (%)*	7 (13 %)	13 (16 %)	0.60 (1)	0.27
Current illicit substance abuse, n (%)*	6 (11 %)	15 (18 %)	0.23 (1)	1.41
Prior antidepressant use, n (%)*	30 (54 %)	16 (19 %)	<0.0001 (1)	17.76
Number of comorbidities (mean ± SD)***	0.54 (+/−0.57)	0.75 (+/−0.75)	0.29 (137)	1.07
Asthma, n (%)**	0 (0 %)	5 (6.1 %)	0.08 (1)	0.08
Chronic obstructive pulmonary disease, n (%)**	1 (1.8 %)	1 (1.2 %)	>0.99 (1)	
Cirrhosis, n (%)*	16 (28.6 %)	31 (37.3 %)	0.28 (1)	1.15
Congestive heart failure, n (%)**	2 (3.6 %)	2 (2.4 %)	0.90 (1)	
Diabetes mellitus, n (%)*	7 (12.5 %)	11 (13.3 %)	>0.99 (1)	0.02
Malignancy, n (%)**	3 (5.3 %)	2 (2.4 %)	0.39 (1)	
Schizophrenia, n (%)**	1 (1.8 %)	0 (0 %)	0.40 (1)	
Stroke, n (%)**	0 (0 %)	3 (3.6 %)	0.27 (1)	

*Chi-Squared test P-value; **Fisher-Exact test P-value; ***Unpaired *t*-test P-value; ***; *SD* standard deviation

**Table 2:** Alcohol-related reasons for index admissions and readmissions

Reasons for Index Admission	Discharged with Antidepressant (*n* = 56)	Discharged without Antidepressant (*n* = 83)	P-value (d.f.)	Test statistic
Alcohol withdrawal, n (%)*	31 (55.4 %)	41 (49.4 %)	0.49 (1)	0.47
Acute alcoholic hepatitis, n (%)*	13 (23.2 %)	23 (27.7 %)	0.55 (1)	0.35
Complications of cirrhosis (ascites, jaundice, encephalopathy), n (%)*	11 (19.6 %)	25 (30.1 %)	0.17 (1)	1.91
Gastritis, n (%)*	6 (10.7 %)	11 (13.3 %)	0.65 (1)	0.20
Acute alcohol intoxication, n (%)**	4 (7.1 %)	13 (15.1 %)	0.19 (1)	
Gastrointestinal bleed, n (%)*	6 (10.7 %)	10 (12.0 %)	0.81 (1)	0.06
Suicidal ideation, n (%)*	7 (12.5 %)	6 (7.2 %)	0.30 (1)	1.10
Chest pain, n (%)**	4 (7.1 %)	6 (7.2 %)	>0.99 (1)	
Other, n (%)**	10 (17.9 %)	7 (8.4 %)	0.10 (1)	
Reasons for Readmission	Discharged with Antidepressant (*n* = 25)	Discharged without Antidepressant (*n* = 39)	P-value (d.f.)	Test statistic
Alcohol withdrawal, n (%)*	10 (40.0 %)	27 (69.2 %)	0.28 (1)	1.15
Acute alcoholic hepatitis, n (%)*	6 (24.0 %)	8 (20.5 %)	0.74 (1)	0.11
Complications of cirrhosis (ascites, jaundice, encephalopathy), n (%)**	4 (16.0 %)	6 (15.4 %)	0.44 (1)	
Gastrointestinal bleed, n (%)**	4 (16.0 %)	7 (17.9 %)	>0.99 (1)	
Gastritis, n (%)**	7 (28.0 %)	3 (7.7 %)	0.04 (1)	
Acute alcohol intoxication, n (%)**	4 (16.0 %)	4 (10.3 %)	0.70 (1)	
Suicidal ideation, n (%)**	2 (8.0 %)	4 (10.3 %)	>0.99 (1)	
Chest pain, n (%)**	0 (0.0 %)	1 (2.6 %)	>0.99 (1)	
Other, n (%)**	1 (4.0 %)	3 (7.7 %)	>0.99 (1)	

*Chi-Squared test P-value; **Fisher-Exact test P-value

The primary outcome (Table 3) showed no significant difference in acute alcohol-related readmissions between patients discharged with versus without a prescription for an antidepressant. Over the study interval, those discharged with a prescription for an antidepressant had between 1 and 4 (median = 1) acute alcohol-related readmissions; for those discharged without a prescription, this range was 1 to 12 (median = 1) readmissions. The most common antidepressant class prescribed by physicians was the selective-serotonin reuptake inhibitors (SSRIs), including citalopram (19.6 %), fluoxetine (19.6 %), sertraline (17.9 %), escitalopram (14.3 %), paroxetine (8.9 %). The next most-commonly prescribed classes were atypical antidepressants [mirtazapine (10.7 %), trazodone (1.8 %)], followed by tricyclic antidepressants [amitriptyline (3.6 %) and nortriptyline (1.8 %)]. The least-prescribed class was SNRIs (venlafaxine = 1.8 %). Due to availability of medications on formulary, SSRIs are most frequently prescribed.

**Table 3:** Independent variables predicting readmission for alcohol abuse (*n* = 139)

Characteristic	% Readmitted	OR (95 % CI)	P-value (d.f.)	Test statistic^a^
Male (*n* = 98)	45.9	1		
Female (*n* = 41)	46.3	1.006 (0.475–2.127)	0.99 (1)	0.00
Smoking (*n* = 44)	43.2	1		
Non-smoking (*n* = 95)	47.4	1.152 (0.549–2.416)	0.71 (1)	0.14
Family History (*n* = 20)	50.0	1		
No Family History (*n* = 119)	45.4	0.823 (0.317–2.137)	0.69 (1)	0.16
Homeless (*n* = 27)	40.7	1		
Not Homeless (*n* = 112)	47.3	1.291 (0.532–3.133)	0.57 (1)	0.32
Prescription for antidepressant upon discharge at index admission				
Yes (*n* = 56)	44.6	1		
No (*n* = 83)	47.0	1.115 (0.561–2.219)	0.76 (1)	0.10

^a^Wald test in the multivariate logistic regression model; *OR* odds ratio, *CI* confidence interval

The multivariate logistical regression model did not show any statistical significance for prescriptions for an antidepressant upon discharge, gender, homelessness, family history, or smoking (Table 3). There was no difference in readmission rates between patients discharged with prescriptions for SSRIs versus other antidepressants (40 % versus 54 %, Chi-square test = 0.58, d.f. = 1, *p* = 0.53).

Patients discharged with a prescription for an antidepressant had a longer LOS (median (IQR): 4 days (4–7.5 days) versus 3 days (2–5 days), Mann–Whitney exact test *p* = 0.10, and higher number of days between index admission and first readmission (median (IQR): 141 days (30–526 days) versus 112 days (27–244 days), Mann–Whitney exact test *p* = 0.28.

## Discussion

Patients with both MDD and AUD who were initiated and discharged from an inpatient stay with an antidepressant prescription had similar readmission rates and intervals between readmissions compared with patients of similar demographics and comorbidities discharged without an antidepressant prescription. Approximately 40 % of patients with both MDD and AUD were discharged with an antidepressant.

A previous meta-analysis showed antidepressant therapy does not significantly reduce depressive symptoms among all patients with both MDD and AUD [27]. The meta-analysis suggested that SSRIs were ineffective. However, TCAs, in particular imipramine and desipramine, and nefazodone, were more effective in treating depressive symptoms.

Prior studies demonstrated treating patients suffering from both MDD and AUD with chlordiazepoxide and imipramine [16, 17], nefazodone [18], sertraline [19, 20], and TCAs [22] did not reduce alcohol consumption. Our analysis did not examine the effectiveness of antidepressants on reducing actual alcohol consumption. However, the lack of an association between discharging patients with an antidepressant and a reduction in future alcohol-related readmission reinforces this observation.

Nevertheless, antidepressant use should not be discouraged in patients with MDD and AUD, as antidepressant therapy is helpful in reducing depressive symptoms in this patient population. Furthermore, unresolved depression is a major factor in relapsing to alcohol use [35]. It is important for healthcare professionals to determine the primary cause of a patient’s alcohol problems, as there are many etiologies. Healthcare providers should consider the strong link between depression and alcohol use disorder; 13.7 % of patients suffering from depression also consume alcohol over a 12-month period [36]. In this population, antidepressants may be warranted since the underlying cause of their alcohol use disorder is depression. Furthermore, a recent meta-analysis indicates that patients who suffer from independent depression with concomitant AUD show greater improvements in their depressive symptoms [37]. Two additional reviews also support this notion that antidepressant therapy is more effective in independent depression [38, 39].

We observed an increase in LOS among patients discharged with versus without an antidepressant prescription. Antidepressants require several weeks to take effect, so no immediate improvement in mood that might facilitate discharge should be expected. Side-effects emerge more quickly. Increasing LOS among patients prescribed antidepressants might be explained by the possibility that such patients may have had more severe depression prompting inpatient evaluation or delaying discharge. While the interval to next admission was not statistically-different between the two groups, the larger interval suggests that antidepressants may be effective for reducing the time to readmission. In a larger study population, a significant difference might be observed.

We investigated whether discharge with an antidepressant prescription modified the effect of literature-identified demographic risk factors for alcohol-related readmissions. Known readmission demographic factors include smoking, family history of alcohol-use disorder, homelessness, and adherence to outpatient alcohol counseling [32, 34, 40]. Our model included these demographic variables except adherence to outpatient alcohol counseling due to the lack of such notation in inpatient medical records. The results (Table 3) from the multivariate model showed no statistical differences in any of the demographic factors for alcohol related readmissions between patients with versus those without the risk factors.

One strength of the current study is the extensive time period examined (2005–2013) and the inclusion of multiple literature-identified risk factors (homelessness, smoking, and family history of alcohol abuse) in the multivariate model. Nonetheless, this study has several limitations. First, it was a single-institution study utilizing a retrospective approach. Retrospective studies may lack data and some patients may have been lost in follow-up. Therefore, our study was relatively small and the patient sample comprised of only patients in the surrounding geographical area. The ability to include various institutions would encompass a more robust and diverse sample generalizable to the overall U.S. population and increase the likelihood of determining whether any variables approaching statistical significance are truly significant. It would also address another limitation by assessing whether patients were admitted to other hospitals, which this study was unable to do. It is also possible that a bias may exist in the group receiving a prescription for an antidepressant. Patients who have more severe depressive symptoms may be more likely to receive a prescription for an antidepressant, and may also be more likely to be readmitted in the future for an alcohol-related event. The current work did not examine the severity of depressive symptoms between the two study groups nor was it able to differentiate if patients had independent depression or substance-induced depression. An additional limitation was lack of means to assess whether patients received other forms of depression therapy (i.e., psychotherapy). The current study did not assess clinical outcomes, such as changes in depressive symptoms or changes in AUD. These therapies are effective in treating both MDD and AUD. Finally, it was not determined whether a patient discharged with a prescription for an antidepressant actually filled the prescription or complied with the medication regimen. This is a concern, as non-compliance with antidepressant therapy is often high due to either the patient experiencing side-effects to the medication or stopping the medication due to the stigma associated with having depression [41]. In addition, economic factors can prohibit patients’ ability to pay for medications [42].

Failure to identify a reduction in alcohol-related readmissions may indicate the complexity of managing dual-diagnosis patients, particularly those with medical comorbidities. Simply prescribing an antidepressant medication to a patient with AUD is unlikely to significantly interrupt the complex chain of events leading to alcohol-related readmissions. Doing so is best accomplished in the context of a medical home model for management of complex, chronic diseases [33]. Such a model would include:

- Reliable, timely access to high-quality primary care
- Insurance coverage for medications
- Referral to and enrollment in substance abuse programs
- Provision of “wrap-around services, including social services, psychotherapy, and substance abuse treatment)
- Integration of physical health, behavioral/mental health, and substance abuse services in a co-management or co-location arrangement

## Conclusions

Discharge with an antidepressant in patients suffering from both MDD and AUD was not associated with lower acute alcohol-related readmission rates. However, antidepressants may still be helpful in patients suffering from independent depression co-occurring with AUD. Healthcare providers should continue to be aggressive and employing strategies in treating AUD in patients also suffering from MDD. Studies with a larger, more generalizable population may shed light on whether antidepressants affect emergency department or inpatient utilization.
